# Preservation of lagging strand integrity at sites of stalled replication by Pol α-primase and 9-1-1 complex

**DOI:** 10.1126/sciadv.abf2278

**Published:** 2021-05-19

**Authors:** Robin van Schendel, Ron Romeijn, Helena Buijs, Marcel Tijsterman

**Affiliations:** 1 Department of Human Genetics, Leiden University Medical Center, Einthovenweg 20, 2333 ZC Leiden, Netherlands.; 2 Institute of Biology Leiden, Leiden University, Sylviusweg 72, 2333 BE Leiden, Netherlands.

## Abstract

During genome duplication, the replication fork encounters a plethora of obstacles in the form of damaged bases, DNA–cross-linked proteins, and secondary structures. How cells protect DNA integrity at sites of stalled replication is currently unknown. Here, by engineering “primase deserts” into the *Caenorhabditis elegans* genome close to replication-impeding G-quadruplexes, we show that de novo DNA synthesis downstream of the blocked fork suppresses DNA loss. We next identify the pol α-primase complex to limit deletion mutagenesis, a conclusion substantiated by whole-genome analysis of animals carrying mutated POLA2/DIV-1. We subsequently identify a new role for the 9-1-1 checkpoint clamp in protecting Okazaki fragments from resection by EXO1. Together, our results provide a mechanistic model for controlling the fate of replication intermediates at sites of stalled replication.

## Teaser

“Primase deserts” in *C. elegans* show how organisms protect genetic information when faced with roadblocks during copying of DNA.

## INTRODUCTION

Stalling of replication forks has been recognized as a strong contributor to genomic instability. Stalled forks are known to occur at sites of DNA damage or thermodynamically stable secondary structures, to result from nucleotide pool imbalances, or to be induced by collisions with the transcription machinery. A great body of work in a variety of biological systems has revealed a network of mechanisms that evolved to secure completion of DNA replication despite these hurdles before cell division. These mechanisms include stabilization of the stalled fork, replisome disassembly, and, in certain contexts, fork reversal to create space for specialized enzymes that repair or resolve the replication impediment. Furthermore, the S-phase checkpoint is activated to prevent mitosis with underreplicated DNA, and eventually, upon completion of repair or bypass, replication is restarted. While recent studies identified a protective role for homologous recombination proteins in protecting nascent DNA strands upstream of stalled forks (*1*, *2*), the biology responsible for maintaining genetic integrity downstream of a replication fork block (RFB) is currently unknown.

## RESULTS AND DISCUSSION

To address the fate of DNA downstream of physiologically relevant RFBs and to assess the consequences for genetic integrity, we monitored mutagenesis at endogenous G-quadruplex (G4) motifs. It was previously demonstrated in *Caenorhabditis elegans* that a single G4 structure under physiological conditions can impose a persistent impediment to DNA replication, requiring the helicase FANCJ/DOG-1 for resolution (*3*–*5*). Failure to unwind the impediment results in a DNA double-strand break (DSB) that requires polymerase θ (POLQ)–mediated end-joining (TMEJ) for its repair (*4*). A distinct mutation profile results in small deletions, typically 70 to 200 base pairs (bp) in size, which have one junction mapping to the stem of the G4 and the other junction ~70 to 200 bp downstream of it. While the nascent strand blocked at the RFB likely defines the proximal deletion junction, it is currently unknown what biology is dictating the location of the junction distal to the RFB, and thus which enzymes suppress excessive DNA loss as the result of underreplication or because of endo- or exonucleic attack on incomplete replication intermediates.

**Fig. 1:**
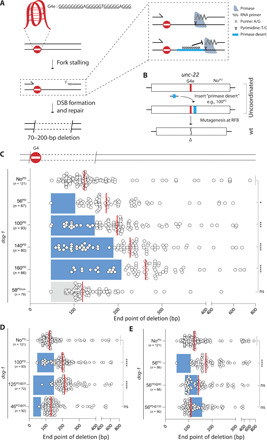
Primase deserts affect mutational outcomes at G4 RFBs. (**A**) A schematic representation of a model explaining G4-induced deletion mutagenesis, where one deletion junction (proximal to the RFB) is defined by the blocked nascent strand, the other (distal to the RFB) by the ssDNA/dsDNA transition of downstream Okazaki fragments. Stretches of pyrimidines, which we termed primase deserts (PDs), are expected to be devoid of primer initiation because primase requires a template pyrimidine to initiate synthesis of the RNA primer. G4e is an endogenous G4 motif inserted in *unc-22*. (**B**) The *C. elegans* genome was engineered to include a G4 sequence at the endogenous *unc-22* gene, which rendered the UNC-22 ORF out-of-frame. The *unc-22*(G4) gene was modified to contain PDs of different length at distinct positions from the G4. These alleles disrupt UNC-22 functionality, yet deletion mutagenesis induced by the G4 can restore the downstream ORF resulting in wild-type moving animals (see fig. S2 for details). WT, wild type. (**C** to **E**) Deletion spectra of *dog-1*–deficient animals with different PDs and a control PD (stretch of pyrimidines) positioned downstream of the G4 motif in *unc-22*. PDs are positioned 47 bp downstream of G4 motif, unless the label contains @ sign, e.g., 125^PD@25^, means a 125-bp PD at 25 bp from the G4 motif. Dots represent independently derived deletion alleles and indicate the position of the distal junctions (in base pairs) relative to the G4 motif set at 0. Blue rectangles indicate the position and size of the PDs, and gray rectangle indicates the position and size of the control PD. Red lines indicate the median. ns, nonsignificant; *P < 0.05, ***P < 0.001, and ****P < 0.0001 by Dunnett’s multiple comparisons test.

We sought to test the idea that DNA synthesis downstream of an RFB limits the size of a vulnerable single-stranded (ssDNA) gap by that act producing an ssDNA/double-stranded DNA (dsDNA) transition point that could define the RFB-distal deletion junction—the size distribution of deletions thus reflecting the ssDNA gap width (Fig. 1A). Previous work indicated that such ssDNA gaps can be converted to DSBs in the next round of replication after the premutagenic lesion has been passed on to daughter cells (*6*). Genetic testing of the logical candidate to initiate DNA synthesis, the DNA polymerase α (pol α)–primase complex, by knockout is impossible given its essential function in genome duplication (*7*, *8*). Instead, we made use of an established biochemical property of this enzyme. RNA primer initiation by the primase subunit is, in fact, not random: All tested eukaryotic pol α-primase complexes use purine as a cofactor to kickstart RNA synthesis and thus require a mandatory pyrimidine template (*8*–*11*). We exploited this necessity by genetically engineering DNA stretches consisting exclusively of purines, which we termed “primase deserts” (PDs) into the *C. elegans* genome downstream of a replication-blocking G4 motif (Fig. 1, A and B). To monitor mutagenic events, we placed a G4 motif flanked by a stop codon in the reading frame of the *unc*–*22* gene, hence generating UNC-22 loss-of-function animals, which move uncoordinatedly (“twitching”; Fig. 1B). G4-induced deletion events that remove the stop codon can lead to restoration of the deletion-tolerant UNC-22 open reading frame (ORF) and reversion to wild-type moving animals, which can easily be isolated from populations of twitching animals. To increase the rate of mutagenesis at replication-blocking G4s, we used animals that lack DOG-1/FANCJ helicase (fig. S1). The deletion spectrum of >50 independently isolated revertant animals is consistent with earlier work: Deletions have one junction flanking the G4 motif immediately upstream and have the other junction 70 to 200 bp downstream of the G4. In addition, these deletions rely on TMEJ (Fig. 1C and fig. S1) (*4*). Next, we inserted PDs (which by themselves do not have predicted G4 folding capability) of different lengths (56, 100, 140, and 160 bp), ~50 bp downstream of the G4 motif (see Fig. 1B and fig. S2 for a schematic illustration) (*12*). None of these deserts affected the reversion rate as compared to the allele without such PD (fig. S1). However, we observed a profound influence on the position of the deletion junction distal to the G4 (Fig. 1C): The vast majority of deletion junctions were found outside the deserts, and the median deletion size shifted proportionally with the PD size. As expected, the position of the proximal junction, likely reflecting the position of the stalled nascent strand, was unaffected (fig. S1). The altered deletion spectrum induced by PDs was completely dependent on the orientation of the desert: Insertion of an inverted sequence at the same location such that a track results exclusively consisting of pyrimidines downstream of the RFB had no effect on the position of the distal deletion junction (Fig. 1C and fig. S2). This outcome suggests that primase activity downstream of an RFB suppresses extensive DNA loss by reducing the ssDNA gap, thereby defining the position where DNA eventually becomes susceptible to end-joining (EJ) activity. Later steps in the biology of G4-induced mutagenesis, i.e., processing of DSBs by TMEJ, appear unaffected as deletions taking out the PDs have microhomology at the junction, occasionally have template insertions, and are completely dependent on functional POLQ (fig. S1).

While junctions are greatly underrepresented in PDs, some were found (Fig. 1C). Although promiscuity of RNA primases, potentially templating on purines, cannot be excluded, these outcomes could also result from priming upstream of the deserts: There are nine pyrimidine bases between the G4 and the deserts, providing templates for primer initiation. Replacement of eight of these nine pyrimidines by purines (the ninth is part of the stop codon downstream of the G4 motif and is essential to the assay) indeed further reduced the number of deletions with a junction within the desert (from 17 to 5%; Fig. 1D). This outcome also suggests (i) that deletion junctions can be located numerous bases away from where a primer starts and (ii) that primase can initiate much closer to an RFB than was suspected on the basis of deletion junctions being >70 bp downstream of the RFB. Priming in close proximity to the stalled replisome may, however, be rare, as analysis of the distributions presented in Fig. 1D points to restricted template availability: The distribution at normal sites (e.g., Fig. 1D, top) resembles that at desert-containing sites if one envisions an occlusion zone from 0 to ~50 bp downstream of the G4. To test this idea directly, we engineered a 46-bp desert immediately adjacent to the G4 motif and found this desert to have no effect on the deletion distribution, strengthening the notion that priming rarely occurs in very close proximity of an RFB, possibly due to steric hindrance by either the RFB or the blocked replisome (Fig. 1D, bottom). Last, we tested how PDs affected the deletion landscape when positioned at 80 and 100 bp downstream of the RFB, where most of the deletion junctions map under unperturbed circumstances. In such a scenario, there is an ample opportunity for primase initiation between the replication stall and the desert. Figure 1E shows a profound and highly indicative disturbance: The deserts split the distributions in two; deletion junctions now predominantly map to either sides of the desert. However, the deserts are not devoid of junctions; for the desert 80 bp from the G4, we found many junctions within the desert, close to its 5′ border. This outcome may be best explained by abundant primase initiation in the region between G4 and desert, while other subsequent biology contributes to the loss of bits of DNA before repair by TMEJ. Later in the manuscript, we will describe one of these contributing activities.

**Fig. 2:**
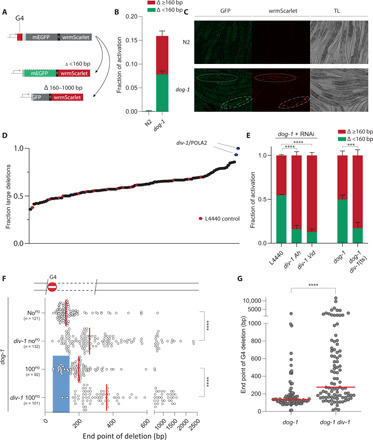
Primase POLA2/DIV-1 activity suppresses DNA loss at G4 RFBs. (**A**) Fluorescent-based reporter able to discriminate G4-induced deletion mutagenesis based on size: Deletions that are <160 bp and bring the downstream ORF in frame with the upstream ATG result in mEGFP and wrmScarlet expression. In-frame deletions that are 160 to 1000 bp will express wrmScarlet exclusively. (**B**) Quantification of reporter activation for ~250 synchronized animals of the indicated genotype. Experiments are performed in triplicate. Error bars denote SD. TL, transmitted light. (**C**) Representative images of fluorescent animals. Long dashes indicate an eGFP- and wrmScarlet-positive animal; short dashes indicate a wrmScarlet-positive animal. (**D**) Ratio of fluorescent animals expressing wrmScarlet exclusively upon RNAi-mediated knockdown of genome stability genes (see table S4). RNAi (L4440) in red. (**E**) Validation of POLA2/DIV-1 by RNAi [Ahringer (*50*) and Vidal (*51*) library clones] in triplicate; and by genetics: *dog-1 div-1*(ts) animals versus *dog-1*. Green indicates animals expressing both mEGFP and wrmScarlet, and red indicates animals exclusively expressing wrmScarlet. Error bars denote SD; ***P < 0.001 and ****P < 0.0001 by *t* test. (**F**) Deletion spectra from the *unc-22* G4 assay, with or without a PD (in blue), for the indicated genotypes. Dots represent independently derived deletion alleles and indicate the position of the distal junctions relative to the G4 motif set at 0. Red lines indicate the median; ****P < 0.0001 by Mann-Whitney test. (**G**) Size representation of deletions at endogenous G4 loci that were found in animals of the indicated genotype. Each dot represents the distal junction relative to the G4 sequence set at 0. ****P < 0.0001 by Mann-Whitney test.

We next aimed to identify genes that contribute to genome stability at sites of stalled replication. Motivated by the suggestion that DNA priming downstream of an RFB affects the degree of DNA loss in a polarized manner, we generated an in vivo reporter system that not only visualizes G4-induced deletion formation but is also able to discriminate between categorically different deletion sizes. We inserted enhanced green fluorescent protein (eGFP) and wrmScarlet (*13*) separated by a 2A sequence (to ensure physical separation of the fluorescent markers upon expression), C-terminal to a small sequence directly downstream of the ATG start codon. This sequence contains a G4 motif and a stop codon to prevent expression of eGFP and wrmScarlet (Fig. 2A). Deletions that take out the stop codon can bring the downstream ORF in-frame with the upstream ATG, resulting in reporter expression. The chosen length of the N-terminal sequence dictates that deletions smaller than 160 bp can lead to expression of eGFP and wrmScarlet, whereas in-frame deletions of 160 to 1000 bp exclusively activate wrmScarlet as GFP-encoding sequence is lost. The reporter functionality and G4 specificity were confirmed by detecting elevated levels of activation in DOG-1–/FANCJ-deficient animals (Fig. 2, B and C): 15% of animals have stochastic patches of somatic cells expressing either eGFP and wrmScarlet (50%) or exclusively wrmScarlet (50%). This 50:50 ratio is in good agreement with deletion size distributions at endogenous G4 motifs. In line with the directionality and asymmetry of deletions, no worms were observed that only expressed eGFP.

We next used these reporter animals to perform a candidate-based RNA interference (RNAi) screen, targeting enzymes that are involved in DNA repair, DNA damage signaling, and DNA replication (table S4). While none of the RNAi clones led to a complete loss of reporter activation, we found two clones that selectively reduced eGFP activation (Fig. 2D): In these knockdowns, 90 to 95% of events exclusively expressed wrmScarlet, indicative of larger deletions. Both RNAi clones target DIV-1/POLA2, which encodes the DNA pol α-subunit B that is part of the DNA pol α-primase complex. We validated our screen by retesting the top 20 RNAi hits in triple and found that only DIV-1 RNAi displayed a consistent increase in wrmScarlet expression (fig. S3). Targeting other members of the DNA pol α-primase complex by RNAi induced embryonic lethality, precluding an assessment of their involvement. Fortuitously, previous genetic studies in *C. elegans* have led to the isolation of a temperature-sensitive (ts) allele of *div-1*/POLA2, which contains a leucine residue instead of an evolutionary highly conserved proline at amino acid position 329 (*14*). We tested animals that are homozygous for this allele at a growth-permissive temperature of 20°C using our reporter and confirmed the RNAi results (Fig. 2E). To obtain a more detailed deletion spectrum, we performed the *unc-22* assay described above and observed a profound effect: The median deletion size of 125 bp in DIV-1 wild-type animals shifted to 262 bp in DIV-1(P329L) animals grown at 20°C (Fig. 2F). When assayed at even lower culturing temperatures (15°C), this shift was less pronounced yet still clearly present (fig. S4), arguing that the P329L mutation affects DIV-1 functionality in a temperature-dependent manner and also in conditions where population growth is seemingly unaffected. In agreement with a proposed role for pol α-primase acting downstream of the RFB, the increase in deletion size can, in its entirety, be explained by nucleotide loss at the RFB distal site (fig. S5). A similar increase was observed in animals where the *unc-22* allele contains a 100-bp PD: Here, the median deletion size shifted from 199 to 362 bp when DIV-1(P329L) animals were assayed (Fig. 2F). To further substantiate the involvement of DNA pol α-primase activity in suppressing DNA loss at RFBs, we assayed G4-induced deletion formation throughout the *C. elegans* genome in an unselected manner: We clonally grew separate populations of *dog-1 div-1*(ts) animals in parallel to *dog-1* controls for 50 generations, after which we sequenced their genomes. We found similar rates for deletion formation at genomic G4 sites in these genetic backgrounds, demonstrating that DIV-1(P329L) expression does not cause elevated fragility or number of G4s (fig. S6). However, and in perfect agreement with the reporter and *unc-22* data, we found a profoundly altered size distribution at G4 loci, with the median deletion size shifting from 125 to 270 bp as a result of altered DIV-1 functionality (Fig. 2G).

We thus found that disruption of the DNA pol α-primase complex via RNAi or genetic mutation leads to a very similar outcome for RFB-induced mutagenesis as the local insertion of sequences that inhibit DNA pol α-primase activity in cis. Together, this provides strong support for the hypothesis that primase activity directly downstream of an RFB protects the genome locally from genetic deterioration. At least two plausible scenarios can be envisaged for recruitment and positioning of the DNA pol α-primase complex at sites of stalled replication: (i) For RFBs located in the lagging strand, Okazaki fragment production by the progressing replisome provides a mechanism for placing RNA primers close to the RFB, and (ii) for RFBs in the leading strand, we favor a prominent role for a converging replication fork, which, upon its approach, will start Okazaki fragment synthesis in close proximity to the RFB. A potential explanation for the observed increased deletion size in DIV-1–compromised *C. elegans* may be a reduced incidence of primer deposition, leading to Okazaki fragments being initiated further away from the RFB. In line with this idea is the recent observation that reduced primase expression in yeast leads to increased Okazaki fragment size (*15*).

**Fig. 3:**
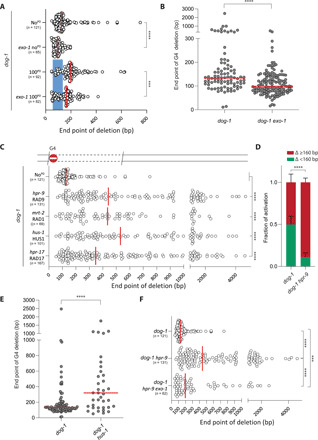
EXO1 activity at G4 RFBs is blocked by the 9-1-1 complex. (**A**) Deletion spectra from the *unc-22* G4 assay, with or without a PD (in blue), for the indicated genotypes. Dots represent independently derived deletion alleles and indicate the position of the distal junctions (in base pairs) relative to the G4 motif set at 0. Red lines indicate the median; ****P < 0.0001 by Mann-Whitney test. (**B**) Size representation of deletions at endogenous G4 loci that were obtained after prolonged culturing of animals of the indicated genotype. Each dot represents the distal junction relative to the G4 sequence set at 0. ****P < 0.0001 by Mann-Whitney test. (**C**) Deletion spectra from the *unc-22* G4 assay for the indicated genotypes. Dots represent independently derived deletion alleles and indicate the position of the distal junctions (in base pairs) relative to the G4 motif set at 0. Red lines indicate the median; ****P < 0.0001 by Dunnett’s multiple comparisons test. (**D**) Quantification of reporter activation (as described in Fig. 2A) for 200 to 300 synchronized L4 animals of the indicated genotype. Green indicates the ratio of animals expressing both mEGFP and wrmScarlet over the total of animals that express one or both fluorochromes, and red indicates the ratio of animals exclusively expressing wrmScarlet. Experiments were performed in triplicate. Error bars denote SD. ****P < 0.0001 by *t* test. (**E**) Size representation of deletions at endogenous G4 loci that were obtained after prolonged culturing of animals of the indicated genotype. Each dot represents the distal junction relative to the G4 sequence set at 0. ****P < 0.0001 by Mann-Whitney test. (**F**) Deletion spectra from the *unc-22* G4 assay for the indicated genotypes. Dots represent independently derived deletion alleles and indicate the position of the distal junctions (in base pairs) relative to the G4 motif set at 0. Red lines indicate the median; ***P < 0.001 and ****P < 0.0001 by Dunnett’s multiple comparisons test.

At present, it is unclear whether Okazaki fragments that are located downstream of an RFB are subjected to exonucleic attack by, e.g., 5′ to 3′ resection enzymes. The presence of deletion junctions within the PDs, in some cases >100 bp away from the nearest primase template, hints toward this DNA processing. One candidate for this activity is EXO1 because of its demonstrated 5′ to 3′ exonuclease activity toward both DNA and RNA in vitro (*16*). We thus generated *exo-1 dog-1* animals and measured G4-induced deletion formation in *unc-22*(G4). Figure 3A shows that the deletion junctions in EXO1-deficient animals are indeed, on average, positioned closer to the G4 motif than in EXO1-proficient animals, the median deletion size being 94 bp instead of 125 bp. A similar reduction in deletion size is observed in alleles carrying a 100-bp PD in addition to the G4 motif: 164 bp versus 199 bp for *exo-1* mutant versus wild type, respectively, arguing that EXO1 activity, on average, removes 30 nucleotides of the 5′ end of newly synthesized DNA at this RFB. To address the generality of this activity, we also determined the sizes of G4-induced deletions that accumulate throughout the genome in *exo-1 dog-1* animals upon prolonged culturing. Compared to EXO1-proficient worms, genomic deletions mapping to G4 loci were, on average, ~40 bp smaller in worms that lost EXO1 activity (Fig. 3B and fig. S6). Our data combined suggest that Okazaki fragment production prevents excessive loss of DNA at RFBs yet are subject to EXO1-dependent degradation.

Since EXO1 is known to perform long-range resection (*17*), we were surprised to observe such a modest loss of only ~30 to 40 nucleotides, which may point to inhibiting factors that we next sought to identify. We focused our attention on the 9-1-1 (RAD9a/HUS1/RAD1) heterotrimeric complex, because it is recruited to sites of stalled replication forks, where it fulfills an essential function in DNA damage-induced checkpoint activation; hence, this complex is also called the checkpoint clamp (*18*, *19*). The 9-1-1 complex structurally resembles PCNA, and an interesting concept here emerges of similar ring-like protein structures providing physical boundaries and functional scaffolds on both ends of an RFB. Consequences of 9-1-1 loss include genomic instability, telomere shortening, and cell death (*20*, *21*). In contrast to mammalian cells, nematodes tolerate a complete loss of 9-1-1, at least for some generations, up to the point that telomeres become critically short, leading to growth arrest, telomere fusions, and animal sterility (*22*–*24*). These delayed detrimental phenotypes provide a sufficient window of opportunity to test the involvement of 9-1-1 complex members in protecting Okazaki fragment deterioration at sites of stalled replication. To this end, we established G4-induced deletion profiles in animals carrying null mutations in HPR-9/RAD9a, in HUS-1/HUS1, and in MRT-2/RAD1. The absence of any single member of the 9-1-1 clamp is known to destabilize the complex, and we therefore expect different null mutations to behave similarly (*22*–*24*). We found that the loss of 9-1-1 had a profound effect on the size of the deletions induced at G4s: While the proximal junction was unaffected, we observed a marked increase in distance and spread of the distal junction, with the median increasing three to four times to 435 to 551 bp and the 10 to 90 percentile ranging from 140 to 1591 bp (82 to 288 bp in 9-1-1–proficient animals) (Fig. 3C and fig. S6). We found an identical outcome by knocking out the clamp loader RAD17 (Fig. 3C) (*25*), arguing that for 9-1-1 to suppress DNA loss at sites of stalled replication, it needs to be physically loaded onto DNA. While the increased loss of DNA is nonsymmetric with respect to the RFB, which argues for a role for 9-1-1 specifically in protecting DNA at the RFB downstream site, we wished to formally exclude the possibility that disturbed repair of consequential DSBs explains our observation: We found that CRISPR-induced DSB repair by TMEJ is not affected by 9-1-1 deficiency (fig. S5). Next, we verified more excessive loss at G4s by 9-1-1 dysfunction in vivo by demonstrating a greatly increased wrmScarlet over eGFP ratio in animals carrying the size-discriminatory G4-deletion reporter (Fig. 3D). Last, we performed whole-genome sequencing of *hus-1 dog-1*–deficient animals after prolonged clonal growth and found the protective effect of 9-1-1 on sites of RFBs acting throughout the genome (Fig. 3E).

We next tested whether 9-1-1 protects the 5′ dsDNA end from EXO1-dependent resection by removing EXO1 from 9-1-1–deficient animals. We observed a significant reduction in the median deletion size in these animals as compared to EXO1-proficient 9-1-1–mutant animals: 196 bp versus 441 bp, respectively (Fig. 3F). While most of the deletions are smaller in an *exo-1* mutant background, large deletions also remain, pointing to previously reported redundancy in processing 5′ DNA ends (*26*).

**Fig. 4:**
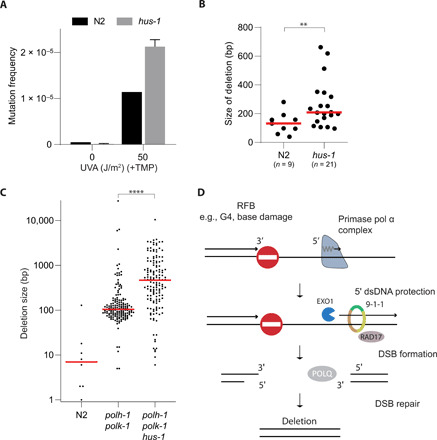
Common molecular and genetic determinants for different RFB types. (**A**) Mutation induction by UV/TMP treatment using the endogenous *unc-22* locus as a mutational target. Wild-type (N2) and *hus-1* mutant animals were either mock-treated or exposed to UV/TMP. Error bar denotes SD. (**B**) Size spectrum of UV/TMP-induced deletion mutations captured at the *unc-22* locus. **P < 0.01 by Mann-Whitney test. (**C**) Size representation of genomic deletions that accumulated in the genomes of the indicated genotype upon prolonged culturing. Red lines indicate median deletion size. ****P < 0.0001 by Mann-Whitney test. (**D**) Tentative model for preserving lagging strand integrity at sites of stalled replication. Upon replication fork stalling at an RFB, the 9-1-1 complex protects newly made Okazaki fragments against nucleolytic degradation by EXO1. In the absence of RFB bypass, the ssDNA gaps will give rise to DSBs that are subject to polymerase θ–mediated end joining (*4*, *6*).

Our data, for which we used G4s as a model substrate, establish a new role for the 9-1-1 complex in limiting loss of genetic information downstream of RFBs. To address the generality of this protective function at RFBs, we extended our investigation to psoralen adducts and spontaneous damage that are dependent on replicative bypass by translesion synthesis (TLS) polymerases (*27*–*29*). Exposing *C. elegans* to trioxsalen (TMP) followed by ultraviolet A (UVA) irradiation leads to replication-blocking psoralen cross-links, which in wild-type animals give rise to deletions randomly spread throughout the genome in the same size range as those accumulating at G4s in *dog-1* animals (*27*). We monitored mutagenesis in wild-type and 9-1-1(*hus-1*)–mutant animals using the wild-type 40-kb-sized *unc-22* gene as a mutational target, isolating UNC-22–deficient worms out of the progeny of exposed hermaphrodites (Fig. 4A). Approximately 50% of the mutants had a deletion disrupting the *unc-22* ORF, and subsequent molecular characterization revealed that these were larger in *hus-1*–deficient animals than in wild-type animals (Fig. 4B). For a third RFB category, we focused on spontaneously occurring DNA lesions that require TLS polymerases η (*polh-1*) and κ (*polk-1*) to be bypassed: Previous work has shown that 50- to 200-bp deletions spontaneously accumulate in the genomes of animals that have impaired TLS activity (*28*). We here performed whole-genome sequencing of animals in which such a TLS defect (*polh-1 polk-1*) is combined with a 9-1-1 defect *(hus-1*) and found that also for this biological context, a 9-1-1 deficiency results in extensive loss of DNA: The median deletion size increased from 104 bp in HUS-1–proficient to 466 bp in HUS-1–deficient animals (Fig. 4C and fig. S6). Together, these data illustrate that 9-1-1 function suppresses extensive loss of DNA downstream of RFBs by counteracting EXO1-dependent nucleolytic degradation of a newly formed 5′ dsDNA segment initiated by the DNA pol α-primase complex. While a mechanism of physical inhibition at the site of the RFB is appealing, it could also be that disturbed 9-1-1–mediated checkpoint activation is causing altered mutagenesis, e.g., by disrupted ATR (ataxia telangiectasia and Rad3-related protein) signaling (*30*).

In this study, we used genetic tools to address the fate of DNA downstream of physiologically relevant RFBs and to identify factors that impact on genetic vulnerability resulting from these RFBs. To study potential deleterious consequences at nucleotide resolution, we made use of two sequence motifs that affect the replication machinery in different ways: While a G4 motif has the ability to fold into a secondary structure that can block the replication fork, hence defining the location of a stalled nascent strand, the newly introduced PD motif prevents the initiation of DNA synthesis and can thus be used to modulate Okazaki fragment positioning in vivo. The combined usage of these two motifs creates an opportunity to temporarily capture an RFB at a fixed genomic position. From the data obtained using this well-defined genetic context, we conclude that (i) Okazaki fragment deposition, either through lagging strand synthesis or brought in by a converging forks, limits the size of vulnerable ssDNA gaps at RFBs; (ii) 5′ ends of Okazaki fragments at RFBs are subject to EXO1-dependent degradation; and (iii) the 9-1-1 complex protects Okazaki fragments against endonucleolytic attack, hence preventing excessive loss of genetic information, meanwhile acting as a damage sensor for checkpoint signaling (Fig. 4D) (*18*, *19*, *25*, *31*, *32*).

Replication stress is considered a universal phenomenon in tumorigenesis (*33*). Arrested forks can evolve into highly toxic and recombinogenic DSBs. It was recently found that mutagenic repair, particularly TMEJ, of replication-associated DSBs results in genomic scars, which are found in disease alleles and in cancer genomes (*34*–*41*). In-depth knowledge on the processing of stalled forks thus contributes to our understanding of genome alterations during cell and organismal evolution, while some of the molecules acting on RFBs are considered promising targets for anticancer therapy (*35*, *37*, *38*, *42*).

## MATERIALS AND METHODS

### *C. elegans* genetics

All strains were cultured according to standard methods (*43*) and grown at 20°C unless otherwise stated. See table S1 for a complete strain list.

### RFB reversion assay

To obtain independent reversion events, single animals were put on 9-cm nematode growth media (NGM) plates (100 to 200) and grown until the food was exhausted. From each wild-type moving animal–containing plate, a single animal was transferred to a new plate to obtain a collection of independently derived deletion alleles. Populations were subsequently lysed in lysis buffer, and DNA was polymease chain reaction (PCR)–amplified with primers surrounding the G4 motif to obtain the deletion products, which were analyzed by Sanger sequencing. The reversion frequency was determined by assaying 75 cultures, starting with placing one animal on a 6-cm plate seeded with 25 μl of the *E. coli* strain OP50. When half of the control *dog-1* populations contained wild-type moving animals, all genotypes were scored for revertants. The reversion frequency is calculated by assuming a Poisson distribution: Reversion frequency = −ln(*P*_0_)/2*n*, where *P*_0_ is the fraction of plates that did not yield revertants and *n* is the number of animals that were screened per plate. Frequencies were determined at least in duplicate and normalized to *dog-1* animals (set to 1).

### Insertion of PDs via CRISPR-Cas9

Plasmids were injected using standard *C. elegans* microinjection procedures. In brief, 1 day before injection, L4 animals of strain XF320 [already containing an inserted G4 sequence (*4*)] were transferred to OP50-containing 6-cm plates and cultured at 15°C. The next day, the gonads of young adults were injected with a solution containing pDD162 [20 ng/μl; Peft-3::Cas9, Addgene #47549; (*44*)], pRS27-29 (20 ng/μl; U6 promoter + sgRNA, Addgene #75026; see table S3 for details on sgRNA sequence), ssODN (20 ng/μl; see table S3 for details), and pBluescript (40 ng/μl). Three to four days after injection, 100 to 200 μl of levamisol (20 mM in M9 salt solution) were added to the plates to find animals with altered *unc-22* alleles. All levamisole-resistant animals were grown to populations for further inspection. *unc-22* mutant progeny animals were analyzed for the presence of a PD.

### Mutagenesis at *dpy-10* locus via CRISPR-Cas9

Plasmids were injected using standard *C. elegans* microinjection procedures. In brief, 1 day before injection, L4 animals were transferred to OP50-containing 6-cm plates and cultured at 15°C. The next day, the gonads of young adults were injected with a solution containing pDD162 [20 ng/μl; Peft-3::Cas9, Addgene #47549; (*44*)], pRS32 (20 ng/μl; U6 promoter + sgRNA; see table S3 for details on sgRNA sequence), pBluescript (60 ng/μl), pGH8 (10 ng/μl), pCFJ90 (2.5 ng/μl), and pCFJ104 (5 ng/μl). Three to four days after injection, mCherry-positive F1 animals were transferred to 6-cm plates. PCRs were performed on animals from plates with germline *dpy-10* mutations in the F2 generation with the following primers: 5′-CAACGAACTATTCGCGTCAG-3′ and 5’-GTGGTGGCTCACGAACTTG-3′. PCR products were send for Sanger sequencing to obtain the specific mutation.

### Creation of the RFB fluorescent reporter

pSR02 (Addgene #69149) was digested with Nhe I and subsequently ligated to remove eGFP to create pRS67. A PCR was performed on pSR02 with a G4-containing primer to create G4::eGFP::T2A and Xba I restriction sites. This PCR product was cloned into pCloneJet and subsequently cut out with Xba I and inserted into Xba-I–digested pRS67 to obtain rps-27:G4::eGFP::T2A::mCherry (pRS68). T2A::mCherry-NLS was then replaced by egl-13::F2A::wrmScarlet::egl-13 (ordered as gBlock) through NEBuilder Hifi DNA assembly to create pRS88: rps-27:ATG::G4::eGFP::egl-13::F2A::wrmScarlet::egl-13. The F2A sequence was added to the design, as an earlier version of this reporter only displayed eGFP and wrmScarlet activation but not wrmScarlet activation alone. We attributed this to degradation of misfolded eGFP protein because of deletions into eGFP. The addition of this sequence solved this issue. pRS88 was cut with Avr II and Hind III and cloned into miniMos vector pCFJ1663 (Addgene #51484) cut with Spe I and Hind III, generating pRS89. N2 worms were injected with a mix containing pRS89 (10 ng μl^−1^), pCFJ601 (50 ng μl^−1^; Addgene #34874), pGH8 (10 ng μl^−1^), pCFJ90 (2.5 ng μl^−1^), and pCFJ104 (5 ng μl^−1^). Five hundred microliters of hygromycin (5 mg/ml) was added to the plates 3 days after injection to select for hygromycin-resistant animals. Plates containing living animals were heat-shocked 7 days after injection for 2 hours at 34°C to counterselect for animals containing extrachromosomal arrays. Animals were then inspected for the presence of both eGFP and wrmScarlet signal. An eGFP- and wrmScarlet-positive strain was obtained, and this strain was subsequently targeted by CRISPR-HDR to switch off the reporter by introducing stops in every frame directly downstream of the G4 motif and to increase the distance between the base of the G4 and eGFP to 160 bp.

### RNAi knockdown

RNAi feeding was performed as previously described (*45*). In brief, we grew RNAi clones against different targets (see table S4) in 2 ml of LB supplemented with ampicillin. The following day, isopropyl-β-D-thiogalactopyranoside (IPTG) was added to the RNAi bacteria to induce dsRNA expression for 2 hours. Six-centimeter NGM plates supplemented with ampicillin and IPTG was seeded with 100 μl of RNAi bacteria and was kept at room temperature overnight. Five L4 animals were transferred to each RNAi-containing plate. After 3 to 4 days, the animals were rinsed off the plate with M9 and inspected for eGFP and wrmScarlet expression.

### Fluorescent reporter readout

Animals of the indicated genotypes were synchronized by hypochlorite treatment, and surviving eggs were hatched in M9 overnight. L1 animals were plated out, and 48 hours later, the animals were rinsed off the plate with M9, sedated with 40 mM NaN_3_, and inspected for eGFP and wrmScarlet expression. To this end, animals were mounted on microscope slides containing dried 2% agarose pads and inspected for eGFP and wrmScarlet expression using a Zeiss Axio imager D2.

### Genomic DNA isolation of MA lines

Mutation accumulation (MA) lines were generated by cloning out F1 animals from one hermaphrodite. All experiments were performed at 20°C. Each generation, three worms were transferred to new plates. MA lines were maintained for 50 generations (*dog-1 exo-1*, *dog-1*, and *div-1*) or 10 generations (*polh-1 polk-1 hus-1*, *dog-1*, and *hus-1*). After 10 or 50 generations, single animals were cloned out and allowed to generate a full population that was used for DNA isolation. To remove bacteria from the sample and from the animal’s intestine, rinsed-off worms were washed three times with M9 and subsequently incubated for 2 hours at room temperature while shaking. After allowing the sample to set down, the supernatant was removed, and the QIAGEN Blood and Tissue Kit was used to extract DNA according to the manufacturer’s protocol with some minor adjustments: 200 μl of ATL buffer and 20 μl of ProtK were added and incubated for 1.5 hours at 60°C in a shaker incubator at 1400 rpm. The samples were spun down for 30 s at 2000 rpm, and the supernatant was transferred to new tubes to prevent blocking of the columns by cellular debris. Then, 5 μl of ribonuclease A (100 mg/ml) was added and samples were incubated for 5 to 10 min, after which 200 μl of AL buffer was first added (mixed thoroughly) followed by 200 μl of EtOH (mixed thoroughly). Spin columns were used and washed with the appropriate buffers, AW1 and AW2, and DNA was eluted in 100 to 150 μl of H_2_O. DNA samples were subsequently prepared according to Illumina’s protocol and sequenced on either HiSeq4000 or NovaSeq.

### Bioinformatic analysis

Mapping of paired-end next-generation sequencing (NGS) reads was performed by BWA-MEM (Burrows-Wheeler Aligner). For each MA line, at least three independently grown samples were analyzed (table S2). For strains that were crossed before growing as MA line, we also sequenced generation 0 to filter out background single-nucleotide variants (SNVs) and copy number variations (CNVs) unrelated to the MA experiment, which may segregate differently in subpopulations. For CNV detection, we made use of Pindel, GRIDSS, GATK, and Manta (*46*–*49*). Only unique events that were supported by at least two callers or called with high confidence (≥5 unique reads supporting the CNV) by a single caller were included in the analysis. Metadata such as homology, topology, and templated insertions were analyzed and categorized using a custom Java program (data file S2). SNV calling was performed by GATK (data file S2).

To annotate the *unc-22* alleles obtained in the *unc-22* G4 RFB assay and UV/TMP assay, a custom Java program was written to extract high-confidence sequences from Sanger sequence files; high-confidence sequence defined as a sequence of >30 nt where all nucleotides have an error probability of <0.05. This sequence is then mapped to a reference FASTA file containing the appropriate *unc-22* allele using k-mer mapping. Differences between the Sanger sequences and the reference are further classified into wild type, SNV, insertion, deletion, or deletion-insertion (delins). Additional information—such as location, homology, and likelihood of templated insertion—was added, leading to output in a TSV format (data file S1).

### Trioxsalen + UVA mutagenesis assay

Animals were synchronized by alkaline hypochlorite treatment (0.5 M NaOH and 2% hypochlorite), and eggs were allowed to hatch overnight. L1 worms were placed on 9-cm NGM agar plates seeded with *Escherichia coli* (OP50) and grown at 20°C. After 48 hours, L4 worms were washed off the plates and treated for 1 hour with TMP (10 μg/ml; Sigma-Aldrich, T6137, stock: 100 mg dissolved in 40 ml of acetone) in M9. Animals were then distributed on nonseeded NGM plates and exposed to UVA irradiation (366 nm; CAMAG 29200 Universal UV LAMP) at a dose rate of 160 μW/cm^2^ (Blak-Ray UV meter, model no. J221). Thereafter, animals were transferred to standard 9-cm OP50/NGM plates (10 P0 animals per plate; 75 plates with treated animals and 50 plates with mock-treated animals). Animals of the F2 generation were washed off the plates with 2 mM levamisole and transferred to six-well plates to facilitate scoring of *unc-22* mutants that are insensitive to the hypercontracting effects of the drug levamisole. For each well, we searched for a levamisole-resistant animal for 120 s. If found, a single levamisole-resistant animal was picked from the well, and homozygous mutants were grown to a full 9-cm plate. Genomic DNA was isolated and analyzed by PCR and Sanger sequencing. The mutation frequency was calculated assuming a Poisson distribution: MF = −ln(*P*_0_)/2*n*, where *P*_0_ is the fraction of plates without reverted animals and *n* is the number of animals that were screened per plate.

## SUPPLEMENTARY MATERIALS

Supplementary material for this article is available at http://advances.sciencemag.org/cgi/content/full/7/21/eabf2278/DC1
